# Role of cardiovascular magnetic resonance imaging in arrhythmogenic right ventricular dysplasia

**DOI:** 10.1186/1532-429X-10-32

**Published:** 2008-06-20

**Authors:** Aditya Jain, Harikrishna Tandri, Hugh Calkins, David A Bluemke

**Affiliations:** 1 Russell H. Morgan Department of Radiology and Radiological Science, Johns Hopkins University, School of Medicine, Baltimore, MD, USA; 2 Division of Cardiology, Johns Hopkins University, School of Medicine, Baltimore, MD, USA

## Abstract

Arrhythmogenic right ventricular dysplasia (ARVD) is a genetic cardiomyopathy characterized clinically by ventricular arrhythmias and progressive right ventricular (RV) dysfunction. The histopathologic hallmark is fibro-fatty replacement of RV myocardium. It is inherited in an autosomal pattern with variable penetrance. ARVD is unique in that it most commonly presents in young, otherwise healthy and highly athletic individuals. The cause of ARVD is not well-known but recent evidence suggests strongly that it is a disease of desmosomal dysfunction. The disease involvement is not limited only to the RV as left ventricle (LV) has also been reportedly affected. Diagnosis of ARVD is challenging and is currently based upon a multi-disciplinary work-up of the patient as defined by the Task Force. Currently, implanted cardioverter defibrillators (ICD) are routinely used to prevent sudden death in patients with ARVD. Cardiovascular MR is an important non-invasive diagnostic modality that allows both qualitative and quantitative evaluation of RV. This article reviews the genetics of ARVD, current status and role of CMR in the diagnosis of ARVD and LV involvement in ARVD.

## Background

Arrhythmogenic right ventricular dysplasia (ARVD) is a progressive cardiomyopathy primarily affecting the right ventricle (RV) and is characterized by fatty/fibro-fatty replacement with myocyte loss, ventricular arrhythmias of left bundle branch block pattern (LBBB) and right heart failure. Increasing evidence suggests that ARVD is a disease of desmosomal dysfunction. ARVD can exist in both sporadic and familial forms with the familial form showing a predominantly autosomal dominant inheritance pattern with variable penetrance. The exact prevalence is unknown, but it is estimated to be 1:5000 in the United States. The clinical onset of ARVD is often delayed to adolescence or early adulthood. It is more commonly seen in males and people engaged in athletics and competitive sports.

ARVD accounts for up to 5% of sudden deaths in young individuals less than 35 years of age in the United States, and 25% of exercise-related deaths in Veneto, Italy [1,2]. The death rate for patients with ARVD has been estimated at 2.5% per year [3]. The main differential diagnosis of ARVD consists of right ventricular outflow tract (RVOT) tachycardia, sarcoidosis, idiopathic dilated cardiomyopathy, isolated myocarditis, adipomatosis cordis, mitral valve prolapse, non-coronary precordial ST-segment elevation (Brugada syndrome) and the Uhl anomaly, which is characterized by a paper-thin RV due to almost complete absence of myocardial fibers [4].

The diagnosis of ARVD is based upon a set of major and minor criteria proposed by the Task Force of cardiomyopathies in 1994 [5]. The diagnostic criteria (Table 1) are based upon a number of diverse genetic, electrocardiographic, pathophysiologic, histopathologic and imaging abnormalities. Patients must have either two major criteria, one major and two minor criteria, or four minor criteria to be labeled as affected with ARVD.

**Table 1:** Criteria for diagnosis of ARVD [5]

I. Global and/or Regional Dysfunction and Structural Alterations*
• **Major**
Severe dilatation and reduction of right ventricular ejection fraction with no (or only mild) left ventricular impairment
Localized right ventricular aneurysms (akinetic or dyskinetic areas with diastolic bulging)
Severe segmental dilatation of the right ventricle
• **Minor**
Mild global right ventricular dilatation and/or ejection fraction reduction with normal left ventricle
Mild segmental dilatation of the right ventricle
Regional right ventricular hypokinesia
**II. Tissue Characterization of Wall**
• **Major**
Fibrofatty replacement of myocardium on endomyocardial biopsy

**III. Repolarisation Abnormalities**
• **Minor**
Inverted T waves in right precordial leads (V_2 _and V_3_) in people aged >12 years, in absence of right bundle branch block

**IV. Depolarization/Conduction Abnormalities**
• **Major**
Epsilon waves or localized prolongation (>110 ms) of the QRS complex in right precordial leads (V_1 _- V_3_)
• **Minor**
Late potentials (signal-averaged ECG)

**V. Arrhythmias**
• **Minor**
Left bundle branch block type ventricular tachycardia (sustained and non-sustained) by ECG, Holter or exercise testing
Frequent ventricular extra-systoles (>1000/24 hours) by Holter

**VI. Family History**
• **Major**
Familial disease confirmed at necropsy or surgery
• **Minor**
Family history of premature sudden death (<35 years) due to suspected right ventricular dysplasia
Familial history (clinical diagnosis based on present criteria)

*Detected by echocardiography, angiography, magnetic resonance imaging, or radionuclide scintigraphy.

The diagnosis of ARVD would be fulfilled by the presence of two major, or one major plus two minor criteria or four minor criteria from different groups.

Important morphological patterns of ARVD manifestation include myocyte loss with fibro-fatty replacement of the ventricular myocardium, focal thinning of ventricular free wall, wall hypertrophy, focal bulging of the RV wall in diastole and RVOT enlargement. Functionally, the disease may result in global or regional contraction abnormalities, RV systolic/diastolic dysfunction, RV dilatation and RV aneurysms. The imaging modalities used for RV assessment in ARVD include conventional angiography, echocardiography, radionuclide angiography, computed tomography (CT) and cardiovascular magnetic resonance (CMR). The standard of reference for the diagnosis of ARVD has been the histo-pathological demonstration of fibro-fatty replacement of the RV myocardium at either surgery or autopsy. Unfortunately biopsy carries risk of perforation and has low sensitivity on account of the segmental nature of fatty infiltration. The major therapeutic decision involved in the care of a patient with ARVD involves the decision to implant an implantable cardioverter defibrillator (ICD). Anti-arrhythmic drug therapy and catheter ablation may be useful to decrease the frequency of sustained ventricular arrhythmias and ICD firing. In case of intractable RV failure or incessant ventricular arrhythmias which are refractory to anti-arrhythmic drug therapy and/or catheter ablation, cardiac transplantation may be an option for treating patients with ARVD.

As novel mutations in desmosomal proteins are being reported, the relationships between genetic mutation and phenotypic expression are beginning to be understood, particularly in the context of CMR diagnosis. This review discusses these relationships and the role of CMR for evaluation of patients with ARVD.

## Genetic basis of ARVD

ARVD is known to occur in both sporadic and familial forms, with familial disease accounting for an estimated 30% to 80% cases [6]. Familial ARVD is primarily autosomal dominant, with several sub-types identified and mapped to distinct genetic loci to date – ARVD1 (14q24.3) [7], ARVD2 (1q42) [8], ARVD3 (14q11-q12) [9], ARVD4 (2q32) [10], ARVD5 (3p23) [11], ARVD6 (10p12-p14) [12], ARVD7 (10q22) [13], ARVD8 (6p24) [14], ARVD9 (12p11) [6], ARVD10 (8q12) and ARVD11 (18q21) [15]. Besides, two autosomal recessive syndromic forms of ARVD – Naxos disease (17q21) and Carvajal syndrome (6p23-24) are also known which involve skin and hair as well as heart [16-18]. An autosomal dominant association of non-epidermolytic palmo-plantar keratoderma, woolly hair, and dilated right ventricle was reported by Tosti et al. in an Italian family [19].

To date, seven genes have been implicated in the dominant and recessive ARVD, including transforming growth factor- β3 (TGFβ3) in ARVD1 [20], cardiac ryanodine receptor (RYR2) in ARVD2 [21] and five desmosomal proteins: desmoplakin (DSP) in ARVD8 [14] as well as Carvajal syndrome [16], plakophilin-2 (PKP-2) in ARVD9 [6], desmoglein-2 (DSG2) in ARVD10 [15], desmocollin-2 (DSC2) in ARVD11 [15] and desmocollin-2 (DSC2) [22] and junctional plakoglobin (JUP) in Naxos disease [17] and also in an autosomal dominant form of ARVD in a German family [23] (Table 2). It is notable that the majority of these genes are involved with cell to cell connection in the region of the cardiac desmosome.

**Table 2:** Candidate genes for ARVD [37]

**Components of the desmosome**
Desmosomal cadherins (desmocollins; desmogleins)*
Desmoplakin*
Emerin
Plectin
Plakophilin*
Tyrosine kinases that phosphorylate desmosomal proteins
**Components of the adherens junction**
α-catenin
β-catenin
γ-catenin (junctional plakoglobin)*
N-cadherin

**Constituents of the gap junction**
Connexin 43

**Others**
Cardiac ryanodine receptor*
Components of dystrophic-glycoprotein complex
Desmin
Laminin receptor-1
Myotonic dystrophy protein kinase-1
Transforming growth factor β3*

*genes that have been definitively implicated in ARVD.

Of the other genes listed above, many are currently under investigation for a potential etiological role in ARVD

ARVD shows polymorphic expressivity with variable clinical expression of PKP2 mutations even among first-degree relatives ranging from a complete lack of symptoms to a severe disease phenotype experiencing sudden cardiac death (SCD)/right or biventricular heart failure. This large heterogeneity in clinical expression can be attributed to factors such as involvement of different desmosomal genes, different mutations within the same gene and effect-modification by other genetic and environmental factors. Dominant ARVD due to PKP2 mutations has also widely been shown to have an age-dependent, variable penetrance which could again be attributed to modifier genes, environmental influences and gender effects [24-29]. In contrast to dominant disease and heterozygous recessive carriers, homozygous recessive forms of ARVD show 100% penetrance by adolescence, with syncope as the usual symptomatic presentation [30].

The genetic patterns of ARVD are complex, with heterogeneity in causative genetic mutations and abnormality within the same gene showing varied patterns of inheritance and phenotypic expression and vice versa. Mutations in DSP have been shown to underlie autosomal dominant and recessive skin disorders, with or without cardiac involvement [31]. Awad et al reported the first case of recessive ARVD caused by a novel cryptic splice mutation in PKP2 [32] and Francis et al reported recessive ARVD with anterior polar cataracts in a consanguineous family from Argentina [33]. Djabali et al reported extensive locus heterogeneity for Naxos syndrome and described exclusion of DSP, JUP, and several other components of the desmosome in two families with Naxos syndrome [34,35]. It is speculated that like other inherited cardiovascular diseases particularly hypertrophic cardiomyopathy and long QT syndrome, a small minority of ARVD cases may have more than one co-existent mutations. Although this is yet to be proven, digenic inheritance with a gene dose effect might however account for many unexplained observations, including relatives with mild but definite disease affliction who are "negative" for the family mutation and variable penetrance and expressivity in families with apparently identical genotype [36].

### Gene expression in the pathogenesis of ARVD

Identification of causative desmosomal mutations has resulted in ARVD to be increasingly viewed upon as a desmosomal disease and has provided insight into its pathogenesis. Mutations affecting components of the cardiac desmosome are believed to disrupt cell-cell adhesion, provoking detachment of myocytes particularly under conditions of mechanical stress. This, in turn, might lead to apoptosis and cell necrosis, predisposing to inflammation and repair by fibro fatty substitution.

Accordingly, early ARVD shows predilection for the regions of heart with relatively thinner walls and high shear stress – the subtricuspid portion, outflow tract, apex and mid-free wall of the RV ("triangle of dysplasia") and the inferior wall septal junction and infero-lateral wall of the LV. Young individuals and athletes are predisposed because of high level of physical activity and resultant greater strain on heart. Gap junction remodeling associated with impaired mechanical coupling at cell adhesion junctions may result in a predisposition to the arrhythmogenicity seen in ARVD. Furthermore, adrenergic stimulation during highly strenuous activity may precipitate arrhythmic events in patients with ARVD. Desmosomal mutations may induce nuclear protein dysfunction as desmosomal proteins are believed to be involved in regulation of transcription. Desmosomal mutations may also heighten the pathogenic role of viral infection speculated in the causation of ARVD [34,36,37].

A pathogenic role of cytoplasmic calcium overload is shown by the association of mutations in RYR2 with ARVD2. This type of ARVD has a very different clinical presentation and is rather distinct from the more common types of ARVD. RYR2 has also been independently implicated in isolated familial catecholaminergic polymorphic VT (CPVT) reflecting possibly different mutations within the same gene and the probable role of effect-modification by individual genetic make-ups and environmental influences in different phenotypic expressions of the same genetic abnormality [38]. Finally, alterations in cytokines such as TGFβ3 may also contribute to disease progression as TGFβ3 is known to induce fibrotic response by stimulating mesenchymal cells and has been shown to modulate expression of genes encoding desmosomal proteins in different cell types and regulate stability of cell-cell junctional complexes [20]. A link between the mutations in the genes involved in ARVD and induction of apoptotic death of myocardiocytes is believed to exist but remains speculative and is yet to be proven [39].

### Role of genetic screening and Task Force diagnostic criteria

There have been a number of studies addressing the issue of prevalence of PKP2 mutations in different groups of ARVD patients [6,25,26,28,40]. Both Dalal et al in the United States [25] and van Tintelen et al in the Netherlands [26] working independently found the prevalence of mutations in PKP2 among unrelated ARVD probands to be 43%. Tintelen et al additionally discovered that the rate of PKP2 mutations was as high as 70% in cases with familial ARVD and no PKP2 mutations were identifiable in cases with non-familial sporadic phenotype. The high prevalence of PKP2 mutations among ARVD patients points towards a potential role of mutation analysis in PKP2 mutation positive ARVD cases and their family members [25]. Cases with PKP2 mutations did not differ significantly from those without PKP2 mutations with regard to clinical characteristics and outcomes, except for earlier age of first clinical presentation and first arrhythmia and more frequent negative T waves in V_2 _and V_3 _[29]. Additionally, patients with a PKP2 mutation undergo ICD interventions irrespective of the classic risk factors determining ICD intervention in ARVD patients [25]. Although molecular genetic analysis is unlikely to contribute significantly to prediction of clinical phenotype and risk stratification, it has potential for family screening and preclinical diagnosis, particularly for patients with early/incomplete disease expression [25,29].

In keeping with variable penetrance and multi-factorial causation of ARVD, the mere inheritance of a mutation previously identified in a proband does not imply a diagnosis of ARVD in a family member, in the absence of clinical diagnostic criteria. Conversely, a fully negative family history could suggest a case of novel mutation or alternatively, a different disease mimicking ARVD such as sarcoidosis, chagas disease, enterovirus infection, chronic right-side myocarditis or sub-acute bartonella infection [39]. The prognostic value of early identification of asymptomatic gene carriers may be limited because a reliable algorithm for primary prevention of sudden death in familial ARVD is lacking and risk stratification in asymptomatic relatives has not been systematically addressed [29,41].

In order to account for the broader spectrum of disease in familial ARVD, it has been suggested that the Task Force criteria be modified such that the presence of any minor criterion in first degree relative of a proven case of ARVD be regarded as clinical disease expression [42,43]. Syrris et al [28] found that the application of modified criteria does expand the diagnostic yield but also may lead to false positives because several family members assumed to be clinically affected were subsequently found to be gene negative. Thus to date the interpretation of minor repolarisation abnormalities in first-degree relatives remains inconclusive.

In summary, a positive genetic result can only be a part of a more comprehensive and multi-disciplinary diagnostic protocol and work-up involving ECG, arrhythmic, morpho-functional, histo-pathological, and clinical/molecular genetic findings [29]. More complete understanding of the underlying genetic basis and the pathogenesis of ARVD will facilitate diagnosis, prognostication and therapeutic decision-making in patients which would be of great significance since adverse clinical outcomes such as SCD occur without premonitory symptoms in many cases.

## CMR of ARVD

**Figure 1:**
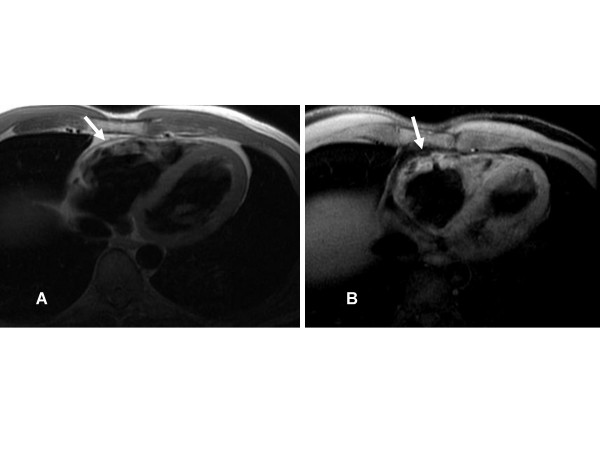
**A**. **Axial black-blood image from a patient with ARVD showing fat infiltration and thinning of the underlying myocardial wall (arrow).B**. Axial fat-suppressed image from the same patient at the same level showing an irregular epicardial surface of the RV due to fat replacement of the RV (arrow).

CMR has emerged as an important imaging modality in the diagnosis and evaluation of patients of ARVD. Among all other clinical imaging techniques, CMR allows clearest visualization of the RV in view of its three dimensional (3-D), multi-planar capabilities with excellent spatio-temporal resolution and improved contrast between blood pool and myocardium [44,45]. CMR can also specifically delineate intra-myocardial fatty infiltration which can be observed as an area of high signal intensity (Figure 1A) on T1 weighted images. CMR additionally offers unique features such as identification of arrhythmogenic foci, velocity mapping of tricuspid flow, objective quantified analysis of RV mechanics using CMR tagging [46] and assessment of ventricular regional, global diastolic and systolic function [44,45]. Unlike other techniques like echocardiography, the 3-D data-set acquired by CMR can be sampled in any plane, making CMR ideal for serial evaluation of the same patient over time [47]. CMR is non-invasive and does not require intravenous contrast or ionizing radiation making it a suitable modality for follow-up in ARVD patients who are frequently young and require a number of repeated examinations. CMR results have been shown to correlate well with RV angiography [48-50], endomyocardial biopsy [51] and echocardiography [50] and can well complement them and other investigations in the diagnosis, work-up and follow-up in this population of patients. It is because of these reasons that CMR appears to be an ideal tool for evaluation of cardiac structure and function in ARVD.

**Figure 2:**
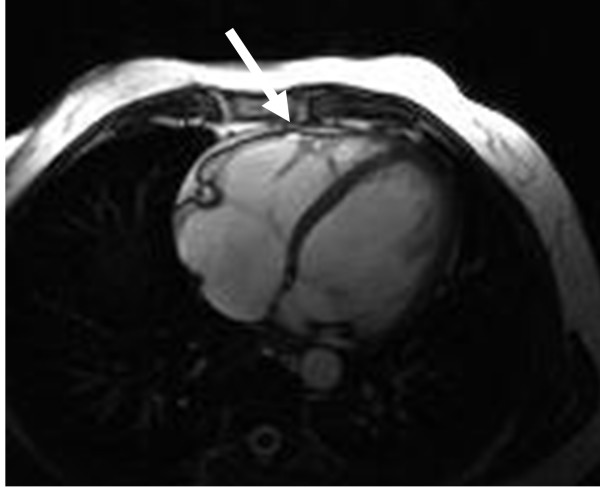
Axial bright-blood image from a patient with ARVD showing a subtle 5 mm aneurysm near the moderator band (arrow).

**Figure 3:**
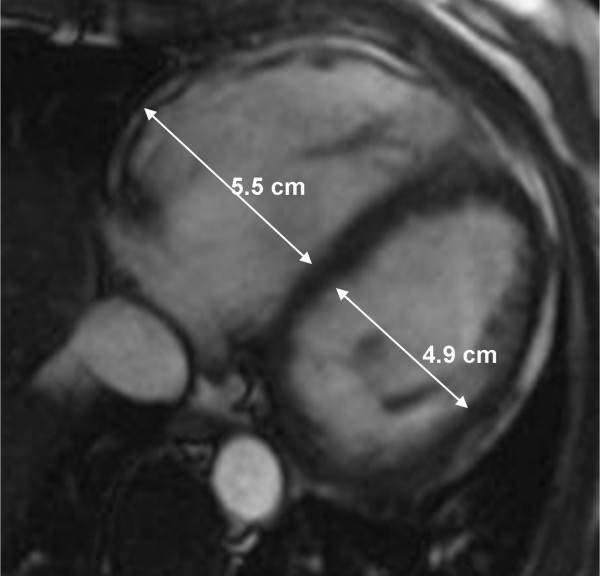
Axial end-diastolic bright-blood image from a patient with ARVD showing RV dilatation. Note the diameter of RV is greater than LV at the mid-ventricular level.

**Figure 4:**
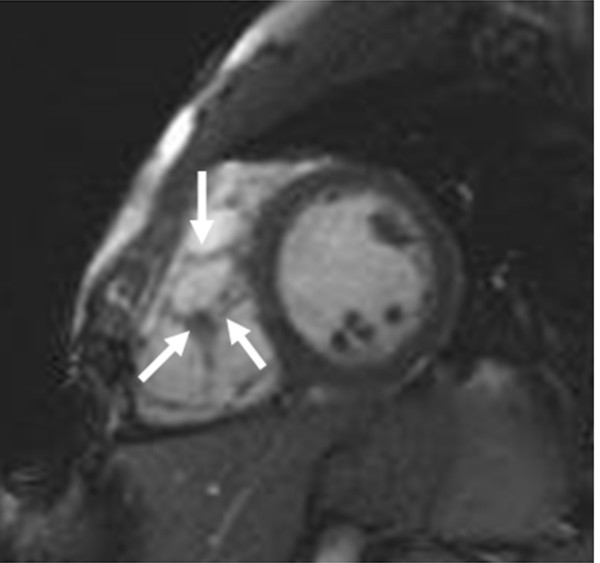
Short-axial bright-blood image from a patient with ARVD showing increased trabeculation in the RV (arrows).

**Figure 5:**
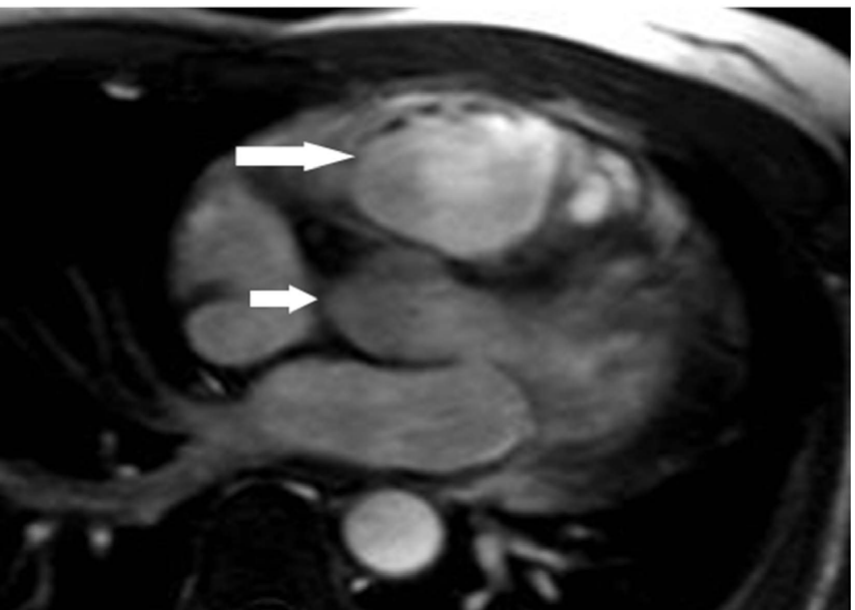
Axial bright-blood image from a patient with ARVD showing enlargement of RV outflow tract (large arrow). Note diameter of RV outflow tract is greater than that of the adjacent aorta (small arrow).

CMR abnormalities in ARVD can broadly be divided into two groups: 1) functional abnormalities which include regional wall motion abnormalities (RWMAs), focal aneurysms (Figure 2), RV dilatation (Figure 3) and RV diastolic/systolic dysfunction 2) morphological abnormalities which include intra-myocardial fatty infiltration (Figure 1), focal wall thinning, wall hypertrophy, trabecular hypertrophy and disarray (Figure 4), moderator band hypertrophy and RVOT enlargement (Figure 5) [52]. Out of these, localized aneurysms, severe global/segmental dilatation of the RV and global systolic dysfunction are considered major criteria while mild global/segmental dilatation of the RV, regional contraction abnormalities and global diastolic dysfunction are considered minor criteria according to the Task Force [5].

Morphologic and functional abnormalities are found most commonly in the "triangle of dysplasia" [44]. Tandri et al systemically described qualitative and quantitative CMR findings in patients meeting Task Force criteria for the diagnosis of ARVD using state-of-the-art CMR [53]. Intra-myocardial fat was noted in a high percentage (75%) of patients who met the Task Force criteria for ARVD, more commonly in the basal regions (RV inflow and outflow) and lateral apex. Trabecular disarray was seen more frequently than wall thinning and aneurysms. RV dimensions and volumes usually are significantly different in ARVD compared to normals and hence useful in the diagnosis of ARVD [54,55].

ECG-gated steady-state free precession imaging (SSFP) pulse sequences (FIESTA, true-FISP, balanced fast field echo) have led to improvements in cine CMR in terms of better contrast between blood and myocardium, better delineation of endocardial borders and more accurate and reliable volumetric measurements of the ventricle as compared to segmented k-space cine gradient echo (GRE) images [e.g. fast low-angle shot (FLASH), fast cardiac-gated gradient-echo (FASTCARD)] [56]. Signal intensity in SSFP is not affected by reduction in velocity of flowing blood in cases of dysfunctional RV as compared to GRE imaging. Schick et al [57] implemented chemical shift-selective sequence to breath-hold cine gradient-echo CMR and found that it improved tissue characterization and differentiation of areas with fibrosis and fatty degeneration from normal myocardial tissue. The use of chemical shift fat suppression (Figure 1B) may also better reveal the true thickness of the RV wall, making the identification of wall thinning or wall hypertrophy in ARVD more reliable [52].

Cardiac-gated spin-echo (SE) pulse sequences have conventionally been used for evaluation of cardiac morphology. CMR black-blood techniques have been developed for cardiovascular imaging to improve segmentation of myocardium from the blood pool. Black blood in the cardiac chambers is achieved by saturation of inflowing blood signal both above and below the acquired volume.

Current black blood CMR techniques (Figure 1A) use a breath-hold fast SE CMR sequence with a dual magnetization preparation pulse (double inversion-recovery) [58-61]. Breath-hold sequences provide end-diastolic images with reduced motion artifacts and improved resolution of myocardial detail. Our group found that CMR obtained using a double R-R fast SE sequence with a short echo time (TE) of 30 msec, an echo train length (ETL) of 24–32 or less if possible, a section thickness of 5 mm, and field of view (FOV) of 24–28 cm were superior to those obtained using other sequences [61].

Black-blood single shot fast SE sequences employ 180 degrees radiofrequency re-focalization pulses preceded by an inversion double pulse associated to pre-saturation pulses. Although these methods substantially reduce motion artifacts and imaging time, the single shot approach frequently results in loss of visualization of the RV. Thus single shot SE is not recommended unless severe arrhythmia is present resulting in failure of other black blood sequences. A full CMR protocol for ARVD is shown in Table 3.

**Table 3:** CMR Protocol for ARVD

*1: Sagittal Scout: any rapid image localizer*
*2: Axial Black Blood Images: double inversion recovery TSE/FSE*
Axial imaging plane provides the best view of the right ventricular anterior wall and the right ventricular outflow tract. Prescribe the axial images starting from the diaphragm to the pulmonary artery.
TR = 2 R-R intervals
TE = 5 msec (minimum-full) (GE); TE 30 msec (Siemens)
Slice thickness = 5 mm
Interslice gap = 5 mm and
Field of view (FOV) = 28 cm
ETL 16–24

*3: Axial SSFP Bright Blood Cine Images*
Same superior to inferior coverage as sequence 2.
TR = 3.5 msec (GE); TR 40–50 msec (Siemens)
TE = minimum
Flip angle = 45–70°
Slice thickness = 8 mm
Interslice gap = 2 mm
FOV = 36–40 cm
16–20 views per segment
Parallel imaging n = 2 is optional

*4: Vertical and long axis cine images (2, 4 chamber view)*
The parameters for this sequence are the same as sequence 3.

*5: Short-axis Black Blood Images*
The parameters for this sequence are the same as sequence 2.

*6: Axial Black Blood Images with Fat Suppression (Optional sequence)*
This sequence is optional and usually adds 10 extra minutes to the total scanning time. Repeat series 2 with chemical selective fat suppression.

*7: Short-axis SSFP cine*
This sequence is prescribed from the four-chamber view. Cover the entire left ventricle. These are performed after gadolinium administration, in order to give time for gadolinium washout. The parameters are identical to sequence 3.

*8: TI scout*
4 chamber view, using TI scout sequences or trial TI times to suppress normal myocardium.

*9: Delayed gadolinium short axis images (10–15 min delay)*
Same slice coverage as short axis cine images.
TR/TE per manufacturer recommendations
TI : optimized to suppress the left ventricle
Flip angle = 20–25°
Slice thickness = 8 mm
Interslice gap = 2 mm
FOV = 36–40 cm
No parallel imaging
Use phase sensitive inversion recovery if available (PSIR)

*10: Delayed gadolinium axial axis images*
Same slice coverage as axial cine images. Pulse sequence same as sequence 9.
TI : optimized to suppress the right ventricle
Use phase sensitive inversion recovery if available (PSIR)

Patient preparation: If the patient is known to have frequent ventricular ectopy, the authors recommend the use of oral Metoprolol 50 mg, 1 hour prior to the procedure provided that the patient has no contraindications. If ventricular arrhythmias are frequent and will substantially impact image quality, the exam should be terminated at this point.

***GADOLINIUM IS ADMINISTERED ACCORDING TO INSTITUTIONAL PROTOCOL (usually 0.15 – 0.2 MMOL/KG)

### Role of CMR in diagnosis of ARVD

Sensitivity and specificity of CMR detection of RV intra-myocardial fat in diagnosis of ARVD is variable, ranging from 22% to 100% [44,48,52,54,62]. Identifying fat can be challenging because the RV is a thin structure and areas of affected myocardium can be quite small. It may be particularly difficult to distinguish pathologic adipose infiltration in areas where adjacent epicardial fat is normally present such as in AV groove and antero-apical portion of RV.

Castillo et al [61] demonstrated that even state-of-the-art clinical CMR protocols using double inversion-recovery fast SE sequences may be limited in both spatial and contrast resolution for detecting fine intra-myocardial structural detail in ARVD. The presence of signal intensity increase seen in T1 weighted images is not specific for fat. Proximity to the surface coil, truncation band artifacts, and various motion-related artifacts may cause projection of high signal intensities onto the myocardium and be mistaken for fat. Similarly, artifacts related to arrhythmia, breathing motion, and blood flow can also produce high signal intensity in the free wall of the RV [63].

The varying sensitivities that have been reported for CMR to detect intra-myocardial fat can be ascribed to patient selection, which includes not only those with overt forms of the disease with extensive morpho-functional alterations and inducible arrhythmias, but also those with concealed forms without global dysfunction and without inducible arrhythmias [62]. Isolated areas of fat replacement have also been reported in elderly patients, long-term steroid use, other cardiomyopathies and in idiopathic RVOT tachycardia, which forms an important differential diagnosis of ARVD [52]. Obesity may be associated with fatty replacement of the RV wall [64]. Macedo et al [65] reported that lipomatous infiltration of RV on cine CMR may occur without global or regional functional abnormalities suggestive of ARVD. Thus RV fat replacement may be a distinct disorder in itself and does not always imply ARVD.

The reliability of detecting increased intra-myocardial T1 signal is probably low. Bluemke et al [55] reported that identification of high T1 fat signal is less reliable than RV measurements or other morphological changes in ARVD.

Abbara at al [63] reported that applying fat-suppressed sequences to conventional fast spin-echo (FSE) imaging leads to an increase in the inter-reader reproducibility and confidence for intra-myocardial fat infiltration, also allowing identification of even subtle to mild degrees of intra-myocardial fatty infiltration (although the significance of such minor fatty changes with respect to their association with ARVD is not clear). Tandri at al [54] similarly found good inter-observer agreement for fat and excellent reliability for other qualitative and quantitative variables among experienced CMR readers by the use of a uniform imaging protocol and application of fat-suppression on breath-hold double-inversion recovery blood suppression pulses. However both Bluemke et al [55] and Tandri et al [54] concluded that the identification of fat within the myocardium is the least specific and least reproducible of any of the other parameters in the CMR evaluation of ARVD. Furthermore, it should be noted that fat signal by imaging tests is not a Task Force criterion for the diagnosis of ARVD.

By the application of CMR, Harper et al [66] confirmed that RV free wall thinning is independently predictive of the presence of ARVD. They quantified RV free wall thinning in ARVD and reported that RV free wall thickness (RVFWT) of less than 5 mm is suspicious for ARVD, between 5 and 8 mm is indeterminate and greater than 8 mm is highly improbable to represent ARVD. Thinning of the RV free wall has been noted as a significant abnormality in ARVD. However, like fat, qualitative evaluation of CMR RVFWT in ARVD may be compromised by thinness of the normal RV free wall, limited in-plane resolution, adjacent high epicardial fat signal and motion artifacts. Like fat, free-wall thinning is also not a part of the Task Force criteria.

Late-enhancement (LE) CMR has the potential to identify myocardial fibro-fatty changes in both RV and LV in ARVD [51,67]. CMR findings suggesting fibrous tissue additionally show correlation with histo-pathological findings and also show association with RV dysfunction and inducibility of VT on electrophysiological (EP) testing [51]. Fibro-fatty variety of ARVD is more common than fatty ARVD. Documentation of fibrous tissue has been suggested to be diagnostically more important than finding fat alone. Fat replacement of RV without fibrosis probably represents a distinct clinico-pathological entity that may not be considered synonymous with ARVD. Fibrous tissue replacement of the myocardium is also believed to be more arrhythmogenic than fat alone [68].

A potential role of CMR lies in its ability to detect early and subtle cases of ARVD which are otherwise insensitive to diagnosis by Task Force criteria, posing a real diagnostic dilemma. In patients with structurally severe disease, all imaging modalities are likely to provide abnormal findings and the ability of CMR to do so therefore does not stand out [69]. CMR can uniquely detect regional and diastolic (right and left) ventricular dysfunction [48,70] which may represent earliest manifestations of ARVD, a continually evolving and progressive disease.

Regional functional abnormalities of RV in ARVD include focal hypokinesis (systolic wall thickening of <40%), akinesis (systolic wall thickening of <10%), dyskinesis (movement of myocardial segment outward in systole), and aneurysms (segments with persistent bulging in diastole and dyskinetic in systole) [52]. Myocardial tissue tagging may potentially improve the sensitivity of CMR to detect regional dysfunction in ARVD because visual analysis is often inadequate due to the complex contraction pattern of the RV [71]. However, tissue tagging is not easily applied due to the very thin RV free wall and poor signal to noise ratio [52].

**Figure 6:**
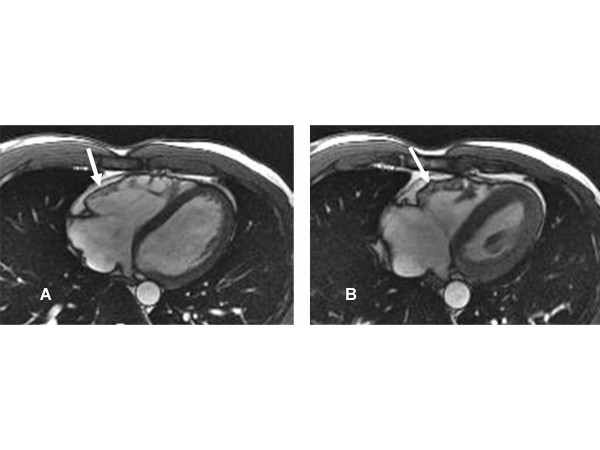
**A**. **Axial end-diastolic bright-blood image from a patient with ARVD. The basal free wall (arrow) appears smooth and regular, with no aneurysms**. **B**. Axial end-systolic bright-blood image from the same patient. A small aneurysm forms at the basal free wall due to dyssynchronous contraction of the RV (arrow).

Regional functional abnormalities have been shown to correlate with areas of signal abnormality. The presence of regional abnormal wall motion associated with signal abnormality is more suggestive of ARVD than either of them alone [52]. Abnormal diastolic dysfunction has been shown to correlate with morphologic abnormalities, indicating that global diastolic dysfunction accompanies more extensive morphologic alterations in patients with ARVD [48,72]. In our experience, regional delayed and dyssynchronous contraction of the RV (mostly at the base) is present in about one-third of phenotypically confirmed cases of ARVD with no other CMR findings of ARVD. We have also found that dyssynchronous contraction of the RV basal free wall may represent an early phenotypic expression of desmosomal mutations (especially PKP2) in the RV in asymptomatic first-degree relatives of ARVD pro-bands. This finding showed good correlation with electrocardiographic minor criteria and inducibility for ventricular arrhythmias (Figure 6).

MR imaging is a useful tool in the evaluation of patients with suspected ARVD. However, it is important to recognize that CMR is not a "gold standard" for the diagnosis of ARVD. But rather, CMR is one of a number of tests which should be employed in the evaluation of patients with suspected ARVD. Other tests which should be performed include a 12 lead electrocardiogram (ECG), signal-averaged ECG (SAECG), Holter monitor, stress test, and echocardiogram. If these tests suggest ARVD, we at our institution, typically recommend biopsy to confirm the diagnosis and exclude other potential etiologies (such as sarcoidosis) mainly in sporadic cases as well as EP study as a part of risk stratification [73]. Great caution must be employed when the only abnormalities which are seen are observed with CMR. In our experience, CMR imaging can result in an over-diagnosis of ARVD. As noted above, many abnormalities which can be seen with CMR, particularly wall thinning and the presence of fat are nonspecific findings. This is important as misdiagnosis of ARVD has great implications not only for the patient but for the family as well. It is extremely uncommon to have an entirely normal 12 lead ECG and have ARVD. Therefore, CMR abnormalities observed in a patient with a normal 12 lead ECG should be interpreted with great caution [74].

Currently, two large clinical trials are under-way that use CMR for ARVD diagnosis. The European ARVD registry [75] attempts prospective validation of criteria for clinical diagnosis of ARVD, evaluation of accuracy of clinical diagnosis and assessment of the natural course of disease [52]. The Multidisciplinary Study of Right Ventricular Dysplasia (U.S. ARVD study) [76] aims to prospectively enroll 100 ARVD patients with 500 first-degree relatives with the following scientific goals: 1) to study a large group of newly diagnosed patients with this uncommon disorder, 2) to elucidate the genetic etiology of this disease, 3) to evaluate treatment of ARVD in these patients. These studies may help better define the role of CMR in ARVD in cases and family members.

## Left ventricular involvement in ARVD

### Introduction

LV involvement in ARVD has been increasingly described, with prevalence reported as 16% [42] to 76% [77]. Hebert et al [78] reported a subnormal LV ejection fraction (EF) by angiography in 20% of a group of ARVD patients. Hunold et al [67] detected LE in the LV in 2 of 6 ARVD patients. Peters and Reil [79] reported that despite a normal LVEF, 40% of their 60 ARVD patients had LV wall motion abnormalities. Bauce et al [31] found LV abnormalities in half of the patients in their study population with echocardiographic evidence of ARVD. Sen-Chowdhry et al [36] demonstrated LV abnormalities with preserved RV function in 40% of their ARVD cohort.

Lindstrom et al [80] demonstrated LV abnormalities in 93% of their ARVD patients by myocardial perfusion scintigraphy and echocardiography. Manyari et al [81] discovered latent LV dysfunction to be present in all cases of ARVD by uncovering it during exercise testing. Evolution of LV involvement in ARVD has been demonstrated in terms of appearance of new abnormalities or worsening of existing abnormalities or both, with progression of RV disease on long-term follow-up.

These cases of early and/or predominant LV involvement in ARVD have brought into question the traditional consideration of LV involvement as only an end-stage complication of progressive RV dilatation and dysfunction in ARVD. Although there is not a complete agreement in literature, LV involvement in ARVD may not stand for a separate disorder, but may be part of a larger disease process extending across the entire heart supporting the adoption of a broader term "arrhythmogenic cardiomyopathy" with appropriate sub-classifications [36].

### Mechanisms of LV involvement in ARVD

The most common variant of ARVD has been postulated to be biventricular with LV involvement from its early stages. The rationale for this is the classification of ARVD as a desmosomal disease and desmosomal genes are expressed in both ventricles. Desmosomal mutations that primarily affect cell adhesion may affect the distensible, thin-walled RV earlier in the course of the disease. LV dysfunction in ARVD may also be secondary to the enlarged and poorly functioning RV because physiologically heart cannot be compartmentalized into functionally independent right and left sides.

### Clinical expression of LV involvement

**Figure 7:**
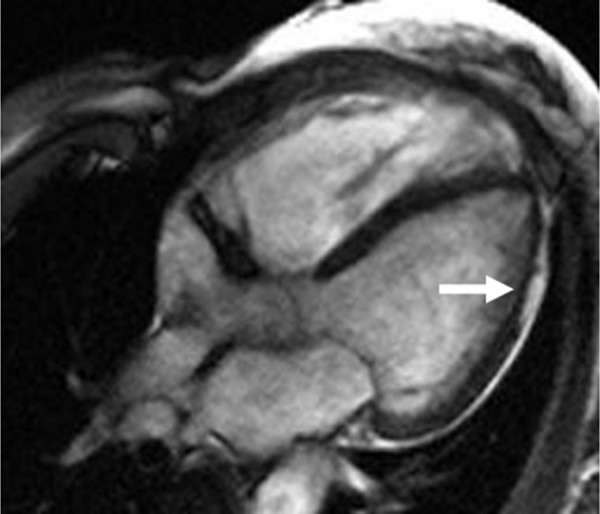
Axial bright-blood image from a patient with ARVD showing a thinned lateral wall of the LV (arrow) due to fatty replacement.

LV involvement in classic ARVD is characterized by extension of T-wave inversion in the ECG lateral leads (V5, V6, L1, aVL), arrhythmia of LV or biventricular origin, and/or isolated LV dilatation and impairment [36]. ARVD fatty/fibro-fatty changes of LV myocardium can extend across varying thickness of myocardial circumference, with a predilection for the sub-epicardial and mid-ventricular wall (Figure 7). Fibro-fatty changes can affect both the septum and more often, the LV free wall, either diffusely or regionally with a predilection for postero-septal and postero-lateral areas [77]. Myocardial replacement of LV by adipose or fibro-adipose tissue, like the RV, is believed to progress inwards from the subepicardium to the trabecular myocardium [82].

Fibro-fatty replacement of the LV shows correlation with myocardial perfusion defects predominantly located in antero-septal and basal posterior segments of the LV, adjacent to the parts of the RV most frequently affected by the disease [80]. Other quantitative and qualitative LV abnormalities that have reportedly been noted in ARVD, like RV, include increased LV end-diastolic pressure, decreased EF, AV block, regional wall motion abnormalities, aneurysms (most frequently at the apex), presence of asynergic areas (most frequently located at the apex or the infero-posterior wall) and dilatation [83].

LV involvement in ARVD appears histologically similar to the RV findings or is only fibrotic [83]. Pinamonti et al found discrete small diverticula of the LV wall on angiography which were thought to represent some localized muscle defect as a component of the underlying ongoing dysplastic process [83]. LV involvement has been detected even in asymptomatic patients and in those with a short duration of symptoms [80,84,85]. LV involvement is more common and more severe in families with several members affected with ARVD [83,86]. LV involvement is associated with increased myocardial mass, inflammatory infiltrates, clinical arrhythmic events and more severe RV wall thinning and heart failure. Frank left-sided heart failure is unusual.

### LV involvement and Task Force criteria

When Task Force criteria were first proposed, minimal LV disease was a stipulation, intended to improve specificity of a then little known disease and ensure distinction from related disorders such as dilated cardiomyopathy. It appears likely that descriptions of LV involvement may be included in subsequent revisions of the diagnostic criteria for ARVD [41].

## Conclusion

ARVD presents a diagnostic challenge on account of the inherent difficulty in its diagnosis using conventional methods, lack of clinical experience due to the rare nature of disease and high frequency of misdiagnosis of the condition. Also, there is no gold standard for its diagnosis and the Task Force criteria which are currently relied upon have never been validated. CMR plays an important role in the diagnosis of ARVD that is both qualitative and quantitative. The recent expansion of the genetic understanding for ARVD will underscore the importance of noninvasive imaging techniques for early phenotypic characterization of this disorder.

## List of abbreviations used

ARVD: arrhythmogenic right ventricular dysplasia; AV: atrioventricular; CMR: cardiovascular magnetic resonance; ECG: electrocardiogram; EF: ejection fraction; ETL: echo train length; FOV: field of view; FSE: fast spin echo; GRE: gradient echo; ICD: implantable cardioverter defibrillator; LBBB: left bundle branch block; LE: late enhancement; LV: left ventricle; RV: right ventricle; RVFWT: right ventricle free wall thickness; RVOT: right ventricular outflow tract; SCD: sudden cardiac death; SE: spin echo; SSFP: steady state free precession; TE: echo time; TI: inversion time; TR: repetition time; TSE: turbo spin echo; WMA: wall motion abnormality

## Competing interests

The authors declare that they have no competing interests.

## Authors' contributions

AJ and HT drafted the initial review article, AJ performed the literature search, HC and DB participated in the article organization, editing and final drafting of the manuscript. All authors read and approved the final manuscript.
